# Clinical Evaluation of Polyglycolic Acid Sheet Application After Partial Glossectomy: A Retrospective Analysis of 284 Consecutive Cases

**DOI:** 10.7759/cureus.115877

**Published:** 2026-09-06

**Authors:** Yasumasa Kakei, Kaito Uryu, Masakatsu Toshikawa, Takumi Imai, Takumi Hasegawa, Masaya Akashi

**Affiliations:** 1 Oral and Maxillofacial Surgery, Kobe University Hospital, Kobe, JPN; 2 Oral and Maxillofacial Surgery, Kobe University Graduate School of Medicine, Kobe, JPN; 3 Clinical and Translational Research Center, Kobe University Hospital, Kobe, JPN

**Keywords:** fibrin glue, partial glossectomy, polyglycolic acid sheet, tongue cancer, wound healing

## Abstract

Objective: To evaluate the clinical outcomes of polyglycolic acid (PGA) sheet application with fibrin glue after partial glossectomy, comparing Neoveil and Neoveil Nano sheets, and to identify factors affecting sheet detachment, postoperative bleeding, and granuloma-like neoplasm (GLN) development.

Study design: We retrospectively analyzed 284 consecutive patients who underwent partial glossectomy with PGA sheet coverage between February 2011 and November 2025. Two sheet types were used: Neoveil (n = 169) and Neoveil Nano (n = 115). The primary outcome was sheet detachment; secondary outcomes were postoperative bleeding and GLN development. Multivariable modified Poisson regression was performed.

Results: The overall engraftment rate was 81.3% (231/284). Neoveil Nano showed significantly higher engraftment (98.3%) than Neoveil (69.8%). Multivariable analysis revealed that Neoveil Nano (risk ratio (RR) = 0.079, 95% confidence interval (CI): 0.019-0.325, P < 0.001) and electrical scalpel use (RR = 0.468, 95% CI: 0.268-0.819, P = 0.008) were independent protective factors against detachment. Postoperative bleeding occurred in 6.3% (18/284). GLN developed in 10.9% (31/284), with a median onset of 53.0 days. Patients with wound-healing-impaired conditions showed a numerically higher GLN rate (17.0% vs 9.7%) and earlier onset.

Conclusions: Neoveil Nano PGA sheets demonstrate superior engraftment compared with conventional Neoveil after partial glossectomy. Electrical scalpel resection also improves engraftment. Sheet detachment was strongly associated with postoperative bleeding, highlighting the importance of sheet adhesion during early wound healing.

## Introduction

Partial glossectomy is a common surgical procedure for the treatment of early-stage tongue cancer and precancerous lesions. Management of the resulting wound is crucial for postoperative outcomes, including pain control, functional recovery, and prevention of scar contracture. While primary closure is often preferred for small defects, larger resections require alternative coverage methods.

Polyglycolic acid (PGA) sheets are biodegradable materials that have been increasingly used to cover oral mucosal defects. When combined with fibrin glue spray, PGA sheets adhere to wound surfaces and promote epithelialization while protecting the wound from oral bacteria and mechanical stimulation. Previous studies have demonstrated the efficacy of this technique, termed mucosal defect coverage with fibrin and PGA sheet (MCFP), in reducing postoperative pain and scar contracture compared with primary closure or artificial dermis [1-4].

Two types of PGA sheets are currently available: Neoveil and Neoveil Nano (GUNZE MEDICAL Ltd., Osaka, Japan). Neoveil Nano, introduced in 2017, features finer fibers and different structural characteristics compared with conventional Neoveil sheets [5]. Recent reports have suggested that Neoveil Nano may provide better engraftment rates in head and neck surgery [2]. However, comparative data specifically for partial glossectomy remain limited.

Clinical complications associated with PGA sheet application include sheet detachment before wound healing, postoperative bleeding, and development of granuloma-like neoplasms (GLNs). A GLN, first characterized by Okuyama et al. as a complication of this technique in the oral cavity, is a clinically evident granuloma-like mass that develops or persists on the wound surface beyond the early healing period and is distinct from normal healing granulation tissue [3]. Understanding the factors that contribute to these complications is essential for optimizing surgical outcomes.

The aim of this study was to evaluate the clinical outcomes of PGA sheet application after partial glossectomy in a large consecutive case series, to compare the performance of Neoveil and Neoveil Nano sheets, and to identify factors associated with sheet detachment, postoperative bleeding, and GLN development. We hypothesized that Neoveil Nano sheets would achieve superior engraftment rates compared with conventional Neoveil sheets, and that patient-related factors affecting wound healing would influence the development of GLN.

## Materials and methods

Patients

This retrospective study included 284 consecutive patients who underwent partial glossectomy with PGA sheet coverage at the Department of Oral and Maxillofacial Surgery, Kobe University Graduate School of Medicine, between February 2011 and November 2025. This study was designed as a retrospective cohort study. The indications for surgery included early-stage tongue cancer (T1-T2), leukoplakia, epithelial dysplasia, and other precancerous lesions. All 284 consecutive patients who met the inclusion criteria during the study period were included in the analysis, and no patients were excluded. There were no missing data for any of the variables examined in this study. Risk factors potentially affecting wound healing were recorded, including diabetes mellitus, history of oral radiation therapy, systemic steroid use, and immunosuppressant use. This study was approved by the Ethics Committee at Kobe University Graduate School of Medicine (approval number: B250265). Because of the retrospective nature of the study, the requirement for written informed consent was waived, and consent was obtained using an opt-out approach in accordance with the requirements of the institutional ethics committee. This study was conducted in accordance with the principles of the Declaration of Helsinki, and the manuscript was prepared in accordance with the Strengthening the Reporting of Observational Studies in Epidemiology (STROBE) statement for observational studies.

Surgical technique

Vital staining with 10% Lugol's solution was used to delineate the resection margins. Excision was performed with a safety margin of 10 mm from the palpable induration and 5 mm from the unstained area [6]. Resection was performed using either a carbon dioxide (CO_2_) laser (n = 81) or electrical scalpel (n = 203). In our institution, the CO_2_ laser was mainly used for precancerous lesions and early-stage (Stage I) cancers, whereas the electrical scalpel was primarily used for resection of squamous cell carcinoma (SCC). After complete hemostasis, solution A of fibrin glue (fibrinogen) was applied to the wound surface, a PGA sheet cut slightly smaller than the wound was affixed, and a mixture of solution A (fibrinogen) and solution B (thrombin) was sprayed over the sheet. Two types of PGA sheets were used: Neoveil (0.15 mm thickness, n = 169) and Neoveil Nano (n = 115). The periods of use for Neoveil and Neoveil Nano were as follows: Neoveil: February 2011-July 2024 (n = 169); Neoveil Nano: September 2020-November 2025 (n = 115). Representative clinical courses using Neoveil and Neoveil Nano sheets are shown in Figures 1, 2, respectively.

**Figure 1:**
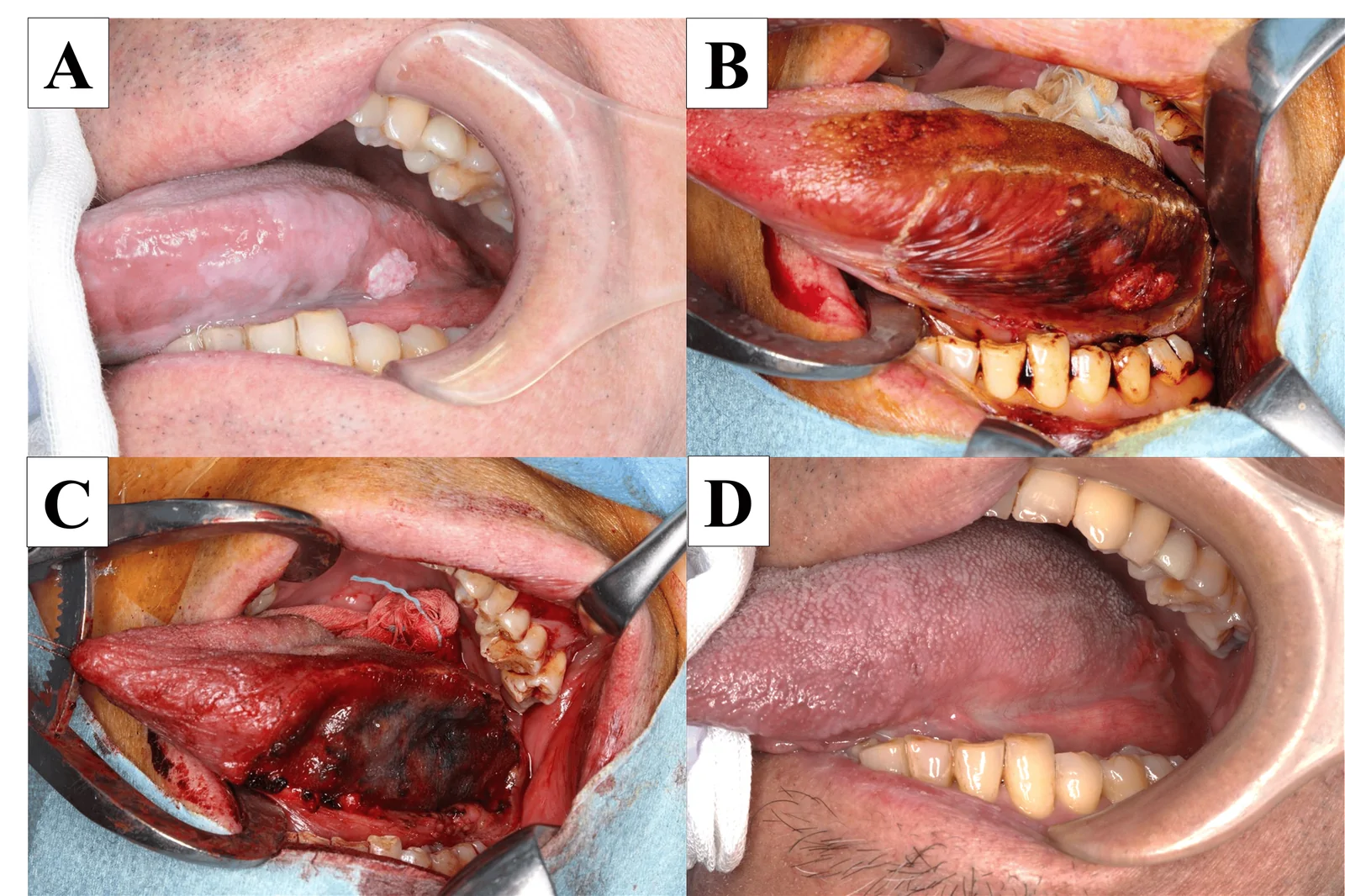
Representative clinical course of partial glossectomy with Neoveil PGA sheet application. (A) Preoperative view showing a lesion on the tongue. (B) Intraoperative view after Lugol’s iodine staining delineating the resection margins. (C) Application of the Neoveil PGA sheet with fibrin glue spray on the raw surface after partial glossectomy. (D) Complete epithelialization of the wound after healing. PGA, polyglycolic acid.

**Figure 2:**
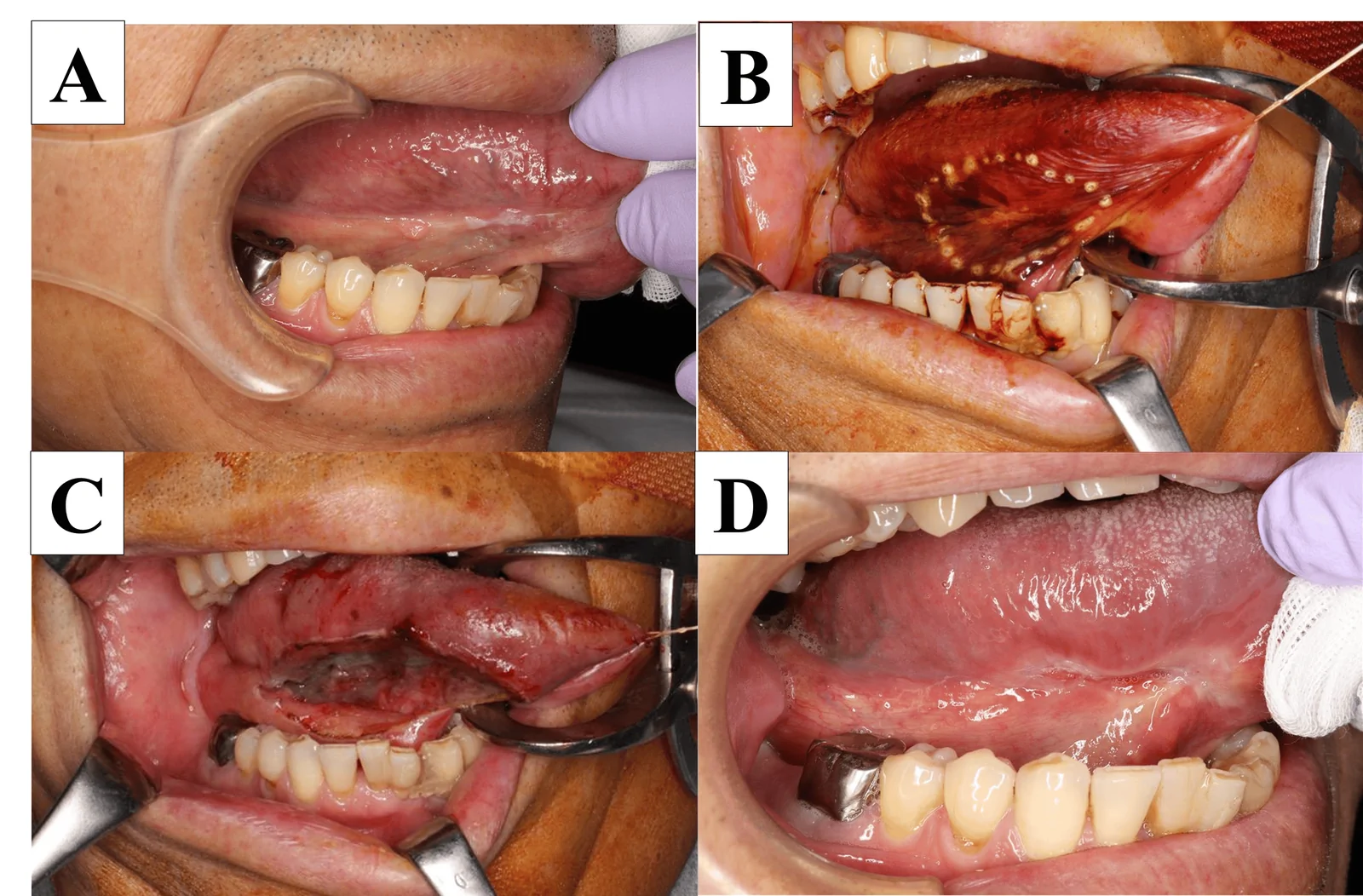
Representative clinical course of partial glossectomy with Neoveil Nano PGA sheet application. (A) Preoperative view showing a lesion on the tongue. (B) Intraoperative view after Lugol’s iodine staining delineating the resection margins. (C) Application of the Neoveil Nano PGA sheet with fibrin glue spray on the wound surface. (D) Complete epithelialization of the wound after healing. PGA, polyglycolic acid.

The excision area was calculated in the same manner as described by Takeuchi et al, approximating the defect as an ellipse using the formula: long diameter × short diameter × π ÷ 4 [1]. 

Outcome measures

The primary outcome was PGA sheet detachment, and the secondary outcomes were postoperative bleeding and GLN development. Sheet detachment was defined as complete loss of the PGA sheet from the wound surface occurring within 14 days after surgery. Because patients undergoing partial glossectomy were typically hospitalized for 10-14 days postoperatively, detachment events were confirmed as in-hospital observations during this period. Postoperative bleeding was defined as any bleeding event documented in the medical records during the postoperative period, including cases managed with compression alone as well as those requiring additional hemostatic procedures such as suturing, coagulation, or surgical intervention. GLN was defined as a clinically evident granuloma-like mass developing or persisting on the wound surface at 14 days or more after surgery, which was distinct from normal healing granulation tissue based on its size, elevation, or progressive growth pattern. This time point was chosen because the acute inflammatory phase of wound healing is expected to subside within the first two weeks after surgery. The onset date of GLN was recorded as the postoperative day when the lesion was first identified during follow-up examination. Risk factors were categorized into diabetes mellitus, systemic steroid use, immunosuppressant use, and history of oral radiation therapy. Diabetes mellitus was defined on the basis of the use of antidiabetic medication. Additionally, patients with diabetes mellitus, steroid use, or immunosuppressant use were grouped as having wound-healing-impaired conditions for subgroup analysis, because these conditions are known to impair the inflammatory and proliferative phases of wound healing through altered immune responses and impaired collagen synthesis. Histopathologic diagnosis was categorized as SCC (including SCC and its variants) versus non-SCC (oral epithelial dysplasia (OED), carcinoma in situ (CIS), and oral intraepithelial neoplasia (OIN)) and included as a covariate in multivariable analyses, because SCC typically requires deeper excision than precancerous lesions. Extension to the floor of the mouth was defined as a resection that extended beyond the tongue across the floor of mouth mucosa toward the mandible lingual gingiva, and was present in 161 cases. The variable was included as a covariate in the multivariable analyses because such extension increases the area of mobile mucosal wound subject to mechanical stress and saliva exposure. 

Statistical analysis

Patients' baseline characteristics were summarized using counts and percentages for categorical variables and basic descriptive statistics for continuous variables. For each outcome, the number of events and corresponding proportions were reported. The cumulative incidence of GLN over the postoperative follow-up period was estimated using the Kaplan-Meier method, with the time origin set as the date of surgery and the event defined as the first clinical detection of GLN. Patients without GLN development were censored at the date of last follow-up.

To explore factors associated with the outcomes, multivariable modified Poisson regression analyses with a robust (sandwich) variance estimator were performed, and associations were quantified using risk ratios (RRs) with 95% confidence intervals (CIs). Covariates included in each multivariable model were selected a priori on the basis of clinical and biological plausibility rather than statistical significance on univariable screening, consistent with recommended practice for confounder selection in observational studies. For multivariable analyses, excision area was modeled per 100 mm² increase to facilitate clinical interpretation of RRs.

When the number of outcome events was small, only univariable analysis was performed, and multivariable analysis was not conducted. All statistical analyses were performed using R version 4.4.2 (R Foundation for Statistical Computing, Vienna, Austria).

## Results

Patient characteristics

The study population consisted of 284 patients (151 male patients, 133 female patients) with a median age of 69 years (range: 23-92 years). Patients with wound-healing-impaired conditions (diabetes mellitus, steroid use, immunosuppressant use, and history of oral radiation therapy) comprised 52 cases (18.3%). These included diabetes mellitus (n = 33, 11.6%), systemic steroid use (n = 8, 2.8%), immunosuppressant use (n = 6, 2.1%), and history of oral radiation therapy (n = 5, 1.8%). The mean excision area was 882 mm². Histopathologic diagnosis was SCC in 194 cases (68.3%) and non-SCC (OED, CIS, or OIN) in 90 cases (31.7%). The resection extended to the floor of the mouth in 161 cases (56.7%). Neoveil sheets were used in 169 cases (59.7%) and Neoveil Nano in 115 cases (40.3%). Patient characteristics are summarized in Table 1.

**Table 1:** Patient characteristics (n = 284) PGA, polyglycolic acid; SD, standard deviation; SCC, squamous cell carcinoma.

Variable	n	(%)
Sex		
Male	151	(53.2)
Female	133	(46.8)
Age (years), median (range)	69	(23-92)
Risk factors present	52	(18.3)
PGA sheet type		
Neoveil	169	(59.7)
Neoveil Nano	115	(40.3)
Resection device		
CO_2_ laser	81	(28.6)
Electrical scalpel	203	(71.4)
Histopathologic diagnosis		
SCC	194	(68.3)
Non-SCC	90	(31.7)
Excision area (mm²), mean ± SD	882 ± 504	

PGA sheet engraftment

The overall engraftment rate was 81.3% (231/284). Neoveil Nano showed an engraftment rate of 98.3% (113/115), while Neoveil showed 69.8% (118/169). Multivariable analysis confirmed that Neoveil Nano use (RR = 0.079, 95% CI: 0.019-0.325, P < 0.001) and electrical scalpel use (RR = 0.468, 95% CI: 0.268-0.819, P = 0.008) were independent protective factors. Histopathologic diagnosis (SCC vs non-SCC) was not significantly associated with sheet detachment (RR = 1.166, 95% CI: 0.707-1.925, P = 0.547) (Table 2).

**Table 2:** Exploration of factors associated with PGA sheet detachment RR, risk ratio; CI, confidence interval; PGA, polyglycolic acid; SCC, squamous cell carcinoma. *P < 0.05, **P < 0.001.

Variable	RR	95% CI	P-value
Sex (female)	0.840	0.531-1.329	0.457
Age	0.993	0.978-1.007	0.322
Risk factors	0.901	0.492-1.649	0.735
Device (electrical scalpel)	0.468	0.268-0.819	0.008*
PGA type (Neoveil Nano)	0.079	0.019-0.325	<0.001**
Excision area (100 mm² increase)	0.988	0.939-1.040	0.651
Extension to floor of mouth	1.303	0.796-2.131	0.292
Histopathologic diagnosis (SCC)	1.166	0.707-1.925	0.547

Postoperative bleeding

Postoperative bleeding occurred in 18 patients (6.3%). Sheet detachment was significantly associated with postoperative bleeding (RR = 5.448, P < 0.001). Among patients with sheet detachment, 18.9% experienced postoperative bleeding, compared with 3.5% in those with maintained sheets.

Granuloma-like neoplasm development

GLN development was observed in 31 of 284 evaluable patients (10.9%). The median time from surgery to GLN detection was 53.0 days (range: 16-95 days; interquartile range: 30.0-61.5 days). The earliest GLN was detected at postoperative day 16, with all cases occurring after 14 days. Eight cases (25.8%) were detected within 28 days, and 23 cases (74.2%) at 28 days or later. The cumulative incidence of GLN, estimated by the Kaplan-Meier method, was 2.8% (95% CI: 0.9-4.7%) at 30 days, 7.7% (95% CI: 4.6-10.8%) at 60 days, and 10.6% (95% CI: 6.9-14.1%) at 90 days postoperatively (Figure 3).

**Figure 3:**
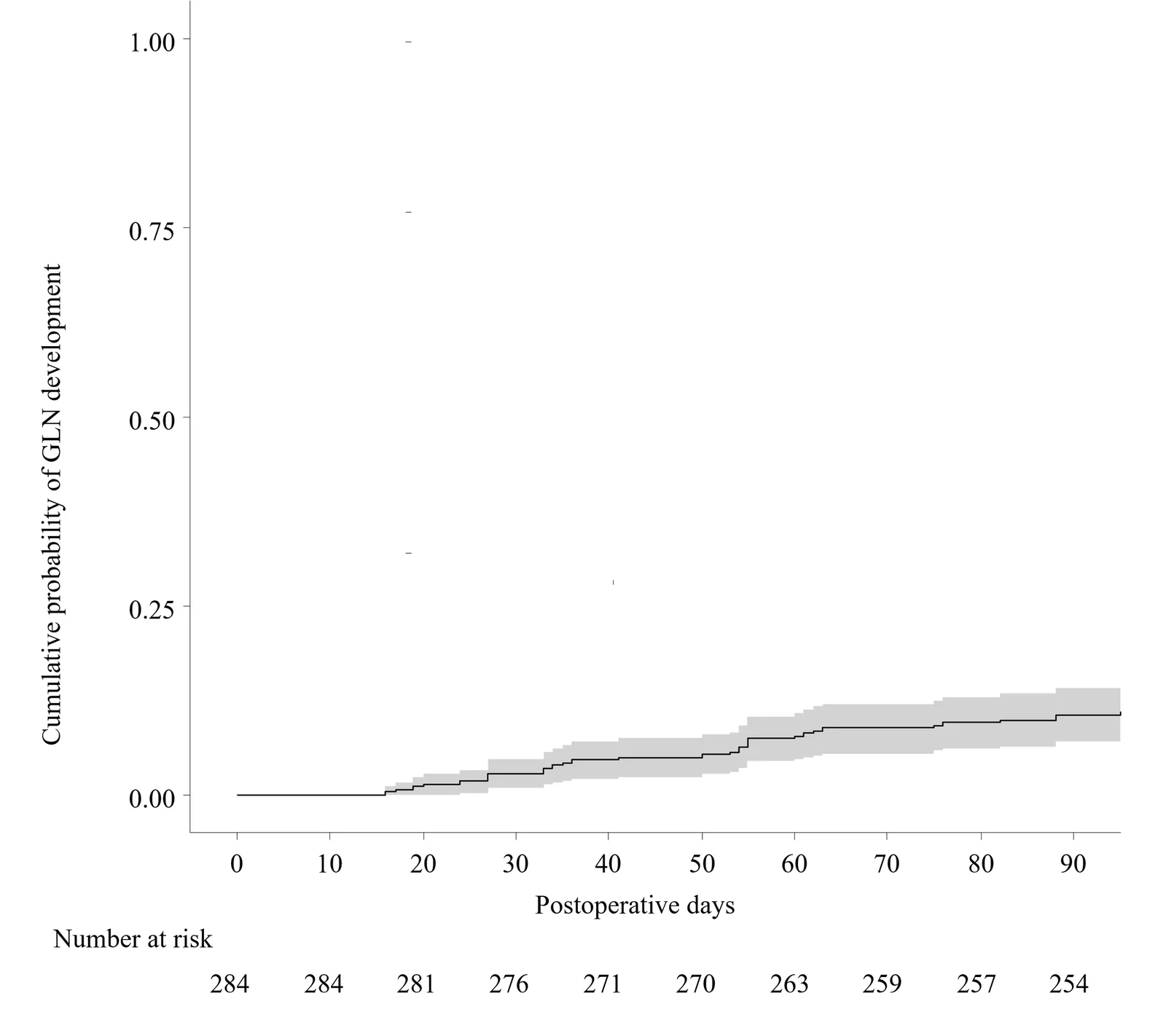
Cumulative incidence of GLN development after partial glossectomy with PGA sheet coverage. Kaplan-Meier curves show the cumulative probability of GLN occurrence over the 90-day postoperative period in 284 consecutive patients. The inset displays the same curve on an expanded vertical scale (0.00-0.15) to better visualize the early postoperative trajectory. The number of patients at risk at each 10-day interval is shown below the graph. PGA, polyglycolic acid; GLN, granuloma-like neoplasm.

When stratified by risk factors, GLN rates were 9.9% (23/232) in patients without risk factors, 15.2% (5/33) in patients with diabetes mellitus, 25.0% (2/8) in those with steroid use, 16.7% (1/6) in those with immunosuppressant use, and 0% (0/5) in those with a history of oral radiation therapy. Patients with wound-healing-impaired conditions showed a numerically higher GLN rate (17.0%, 8/47) compared with those without (9.7%, 23/237), although this difference did not reach statistical significance (P = 0.196 by Fisher’s exact test). The median onset of GLN was earlier in patients with wound-healing-impaired conditions (38.5 days) compared with those without (54.0 days), although the difference was not statistically significant (P = 0.190 by Wilcoxon rank sum test). In the multivariable analysis, there were no associations with GLN development detected for PGA sheet type, sheet detachment status, histopathologic diagnosis, and excision area.

## Discussion

To our knowledge, this is the largest single-institution series to date evaluating PGA sheet application after partial glossectomy and the first to directly compare the engraftment performance of conventional Neoveil and Neoveil Nano sheets in this anatomical setting. Three principal findings emerge: (1) Neoveil Nano achieved a markedly higher engraftment rate than conventional Neoveil (98.3% vs 69.8%); (2) electrical scalpel resection was independently associated with improved sheet engraftment compared with CO_2_ laser; and (3) sheet detachment was strongly linked to postoperative bleeding, underscoring the importance of stable sheet adhesion during the early healing period.

The overall engraftment rate of 81.3% in the present cohort exceeds the 75% rate reported by Kondo et al. for tongue lesions [2]. More notably, the Neoveil Nano subgroup achieved a near-complete engraftment rate of 98.3%, which substantially surpasses previously published results in head and neck mucosal defect coverage and indicates that sheet detachment can largely be eliminated with appropriate material selection.

The superior performance of Neoveil Nano is likely attributable to its structural characteristics. The finer PGA fibers and larger inter-fiber distances of the Neoveil Nano fabric may facilitate earlier and more uniform cellular infiltration while limiting excessive inflammatory granulation [5]. Although designated “Nano,” the fibers of this fabric are on the micrometer rather than the nanometer scale (approximately 3 μm in diameter, vs approximately 16 μm for conventional Neoveil), and the inter-fiber distance is correspondingly larger (approximately 40 μm vs approximately 5-20 μm) (GUNZE MEDICAL Ltd, personal communication). In addition, its thinner profile may reduce the likelihood of food impaction and mechanical peeling, which are particular concerns on the highly mobile tongue surface. The independent protective effect of electrical scalpel use likely reflects more precise wound surface preparation and superior intraoperative hemostasis compared with CO_2_ laser, both of which favor immediate fibrin-mediated adherence of the PGA sheet. It should also be noted that, at our institution, Neoveil was used from 2011 to 2024, whereas Neoveil Nano was introduced in 2020. The higher engraftment rate observed with Neoveil Nano may therefore have been influenced in part by the surgical team's accumulated experience and increasing familiarity with handling PGA sheets gained during the earlier Neoveil period.

The postoperative bleeding rate of 6.3% in this study is consistent with the 7% reported by Okuyama et al [3]. Notably, sheet detachment was strongly associated with postoperative bleeding (RR = 5.448; 18.9% vs 3.5%), in agreement with prior reports linking premature sheet loss to bleeding events after resection of oral and oropharyngeal mucosal lesions [4]. Together, these data emphasize the clinical importance of maintaining sheet adhesion throughout the critical early phase of wound healing and provide an additional argument in favor of materials, such as Neoveil Nano, that achieve more reliable adherence.

GLN development occurred in 10.9% of patients in our cohort, slightly higher than the 8% reported by Okuyama et al [3]. Although their single-site analysis identified tongue application as a risk factor, our tongue-limited cohort did not allow replication of that finding. Kouketsu et al. [7] similarly reported conspicuous granulomatous proliferation requiring excision in two of 24 partial glossectomy cases, suggesting that GLN is a recognized, if infrequent, complication of the MCFP technique on the tongue across different fibrin glue formulations. While the association between wound-healing-impaired conditions and GLN did not reach statistical significance (P = 0.196), the numerically higher rate in this subgroup (17.0% vs 9.7%) suggests that patient-level factors warrant further attention.

We defined GLN as a granuloma-like mass identified at 14 or more days postoperatively to distinguish pathological proliferation from physiological granulation. This cutoff is biologically grounded: macrophage accumulation peaks at day 7 and subsides by day 14, by which time the proliferative phase is well established. Our data support this threshold, as the earliest GLN was detected on postoperative day 16. The temporal distribution (16-95 days; median 53.0 days) was earlier than the approximately three-month average reported by Okuyama et al. [3], which may reflect our prospective, protocol-based follow-up and standardized recording of onset dates. Importantly, 25.8% of GLNs were detected within 28 days, indicating that vigilance for early-onset GLN should not be deferred to late postoperative visits.

When examined by individual risk factor, GLN incidence was highest with steroid use (25.0%), followed by immunosuppressant use (16.7%) and diabetes mellitus (15.2%), whereas no GLN was observed in patients with a history of oral radiation therapy. Although these subgroup comparisons did not achieve statistical significance, likely owing to limited subgroup sizes, the aggregated wound-healing-impaired group exhibited both a higher GLN rate (17.0% vs 9.7%) and earlier onset (median 38.5 vs 54.0 days). Mechanistically, diabetes mellitus impairs macrophage function, prolongs the inflammatory phase, and reduces collagen synthesis, whereas steroids and immunosuppressants suppress immune cell recruitment and cytokine production; each of these alterations may permit aberrant tissue proliferation in response to a foreign material. The absence of GLN among irradiated patients may conversely reflect radiation-induced fibrosis and reduced vascularity, which could blunt excessive granulation. Of note, animal studies have demonstrated strong fibroblast growth factor 2 (FGF-2) expression in both epithelial and connective tissue layers at one week after partial glossectomy in rabbits treated with the MCFP technique [8], indicating that fibrin-mediated fibroblast proliferation and active granulation are intrinsic to wound healing under PGA sheets. GLN may therefore represent an exaggerated or persistent form of this otherwise physiological response, particularly when normal resolution is delayed in patients with impaired healing capacity.

These observations align with established mechanisms of PGA-related inflammation [9]. PGA undergoes hydrolysis into glycolic acids, lowering local pH and provoking inflammatory responses. Glycolide, a degradation product, activates the complement pathway and increases C5a, recruiting macrophages and neutrophils to the wound site [10]. The resulting cytokine milieu (TNF-α, IL-1β, IL-6, and IL-8) sustains local inflammation [9], while PGA degradation has also been associated with an increased number of multinucleated giant cells and a reduced proportion of M2 macrophages, indicating a proinflammatory shift [11]. These pathways provide a plausible mechanistic basis for GLN formation in our cohort, particularly in patients in whom the inflammatory-to-proliferative transition is already disrupted. Strategies to modulate this response, such as incorporation of sodium tripolyphosphate to enhance IL-10 expression [12], warrant further investigation in oral mucosal wound management.

These mechanistic considerations also frame the development of next-generation biomaterials. Emerging materials seek to add active functionality to passive coverage, including anti-inflammatory effects, antimicrobial activity, and sustained drug release [9]. PLGA (poly(lactic-co-glycolic acid))-based nanoparticle systems, bioactive glass-hydrogel composites, and electrospun mucoadhesive membranes capable of delivering therapeutic proteins (including anti-TNF-α) have shown promise in preclinical models, and adhesive immunomodulatory patches, including microneedle-based platforms for localized checkpoint inhibitor delivery, suggest future applications for post-resection immunosurveillance in oral cancer [9]. Five core requirements have been proposed for such materials: flexibility, adhesion, infection resistance, controlled drug release, and customizability through 3D- and AI-based design [9]. Multifunctional materials capable of actively modulating the inflammatory environment and promoting functional mucosal regeneration would directly address the principal limitations identified in the present study.

The clinical relevance of PGA sheets extends beyond head and neck surgery. A systematic review and meta-analysis by Peng et al. demonstrated that prophylactic PGA sheet placement significantly reduced both post-endoscopic submucosal dissection (ESD) bleeding (RR = 0.35; 95% CI: 0.19-0.64; P < 0.001) and esophageal stricture (RR = 0.46; 95% CI: 0.27-0.79; P = 0.005) [13]. Proposed mechanisms include protection from exogenous stimuli, suppression of local inflammation, scaffolding for cell adhesion and tissue regeneration, and reduction of scar contracture, which largely overlap with those relevant to oral mucosal coverage. By contrast, on exposed bone surfaces, PGA sheets are reported to adhere firmly without early detachment and to undergo uniform absorption concurrent with epithelialization [14]. The contrast among mobile soft tissue, gastrointestinal mucosa, and bone substrates highlights the influence of anatomical site, mechanical environment, and epithelialization pattern on PGA sheet performance, and underscores the need for site-specific optimization of these biomaterials.

The present findings have direct implications for clinical practice. First, Neoveil Nano should be preferred over conventional Neoveil for partial glossectomy defects whenever available, given its substantially higher engraftment rate and the strong association between sheet detachment and postoperative bleeding. Second, electrical scalpel resection appears preferable to CO_2_ laser when sheet engraftment is a priority, although the choice of energy device should remain individualized to the surgical context. Third, in patients with diabetes mellitus, systemic steroid use, or immunosuppressant use, clinicians should anticipate an elevated and earlier-onset risk of GLN, and structured follow-up at approximately three to four weeks postoperatively may facilitate timely detection.

Several limitations should be acknowledged. First, the retrospective single-institution design limits external generalizability and precludes formal causal inference. Second, the study spans more than 14 years, during which surgical technique, postoperative care, and the choice of resection device evolved; the introduction of Neoveil Nano at our institution in 2020 introduces temporal confounding that cannot be fully resolved by multivariable adjustment. Third, the small number of events in some risk-factor subgroups (e.g., systemic steroid use, immunosuppressant use, history of oral radiation therapy) limited the statistical power to detect associations with GLN, and the wide 95% CIs in these analyses should be interpreted accordingly. Fourth, GLN was diagnosed clinically without routine histopathological confirmation, although the prolonged time-course and clinical phenotype were consistent with prior definitions [3]. Fifth, functional outcomes such as speech, swallowing, and scar contracture were not systematically assessed, leaving the relationship between sheet engraftment status and longer-term functional recovery to be established. Prospective, multicenter studies with standardized assessment protocols are warranted to confirm these findings and to clarify the role of patient-related factors in GLN development.

## Conclusions

These findings support the preferential use of Neoveil Nano sheets combined with electrical scalpel resection for partial glossectomy defects, as this combination optimizes sheet engraftment and may reduce postoperative bleeding risk. Close postoperative surveillance is recommended for patients with wound-healing-impaired conditions, who appear more susceptible to GLN development.
